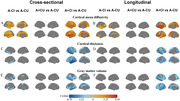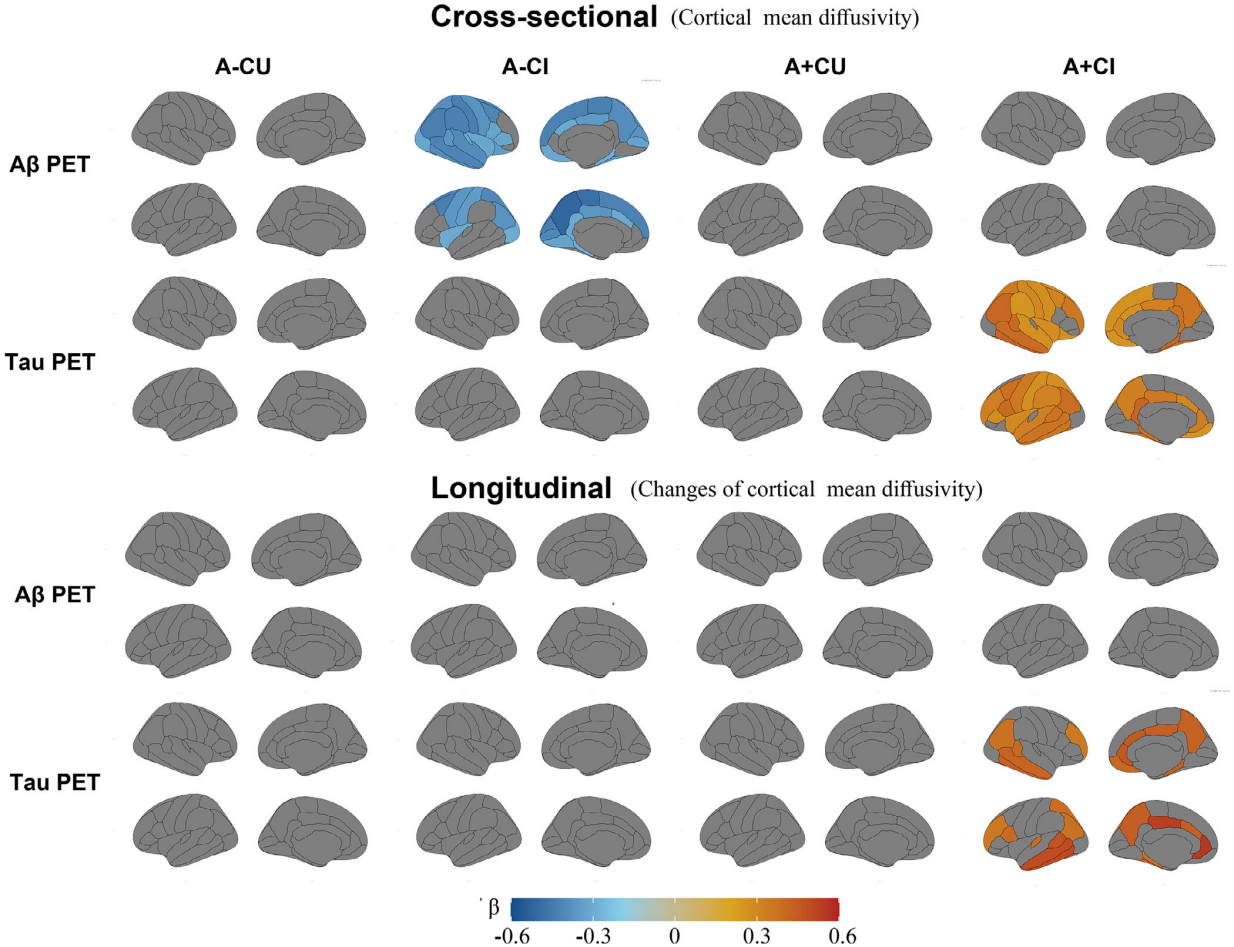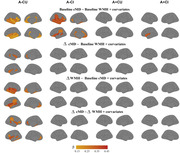# Association of microstructural changes with vascular disease and Alzheimer’s Disease

**DOI:** 10.1002/alz.090271

**Published:** 2025-01-09

**Authors:** Pan Sun, Zhengbo He, Anqi Li, Yalin Zhu, Yue Cai, ADNI, Tengfei Guo

**Affiliations:** ^1^ Institute of Biomedical Engineering, Shenzhen Bay Laboratory, Shenzhen, Guangdong China; ^2^ Tsinghua Shenzhen International Graduate School (SIGS), Tsinghua University, Shenzhen China; ^3^ Alzheimer’s Disease Neuroimaging Initiative, http://adni.loni.usc.edu/, CA USA; ^4^ Institute of Biomedical Engineering, Peking University Shenzhen Graduate School, Shenzhen China

## Abstract

**Background:**

Non‐invasive diffusion‐weighted imaging (DWI) can be used to assess cortical microstructure through cortical mean diffusivity (cMD). However, it is still unclear about the dynamic changes of cMD, as well as how it relates to vascular disease and Alzheimer’s disease (AD).

**Method:**

We identified 318 cognitively unimpaired (CU) and 305 cognitively impaired (CI) participants with DWI image, β‐amyloid (Aβ) positron emission tomography (PET) image from the Alzheimer’s Disease Neuroimaging Initiative cohort. The 623 participants were separated into A‐CU, A‐CI, A+CU, and A+CI groups. Microstructural changes reflected by cMD were processed with a surface‐based diffusion tensor imaging (DTI) approach. Aβ PET was processed using cortical summary composite standardized uptake value ratio (SUVR) and converted to the Centiloid following the standard equation from the ADNI website. Tau PET SUVR was extracted in the composite temporal‐metaROI. T2‐flair‐based magnetic resonance imaging (MRI) was utilized for white matter hyperintensity (WMH) total number calculation with an in‐house developed algorithm and toolbox. T1 MRI image was processed using Freesurfer, and the cMD value in the 68 Desikan‐Killiany atlas regions of interest was extracted. Linear mixed effect (LME) models were used to calculate slopes of cMD for all participants with longitudinal DWI and WMH data. Generalized linear models (GLM) were used to compare baseline and slope of cMD among subgroups, adjusting for age, sex, APOE‐ε4, and education.

**Result:**

Compared to the commonly used macrostructure indicators of cortical thickness and gray matter volume, the micro‐structural indicator cMD exhibited high sensitivity of group‐level alteration crosse‐sectionally and longitudinally for both vascular disease and AD (Figure 1). cMD was negatively correlated with Aβ in the A‐CI group but positively related to tau in the A+CI group. Tau PET was associated with faster increases of cMD only in the A+CI group (Figure 2). In contrast, cMD and WMH were linked with each other in both cross‐sectional and longitudinal datasets in Aβ negative individuals (Figure 3).

**Conclusion:**

The findings suggest that cortical microstructure alternation reflected by cMD may be a promising biomarker for neurodegenerative disease compared to the cortical thickness and gray matter volume, particularly for vascular disease.